# One-year mortality among adults with advanced HIV in sub-Saharan Africa: a systematic review and meta-analysis

**DOI:** 10.1097/QAD.0000000000004431

**Published:** 2026-01-22

**Authors:** Thomas C. Scheier, Keisha De Gouveia, Mark E. Engel, Ameer S.-J. Hohlfeld, Alex Cen, Anne Berhe, Sabrina Fan, Jeffery Li, Shakeap Elliott, Nathan Ford, Graeme Meintjes, Dominik Mertz, John Eikelboom, Sean Wasserman

**Affiliations:** a Population Health Research Institute, Hamilton, Ontario, Canada; McMaster University and Hamilton Health Sciences, Hamilton, Ontario, Canada; b Clinical Research Centre, Faculty of Health Sciences; c Cape Heart Institute, Faculty of Health Sciences, University of Cape Town; d Cochrane South Africa, South African Medical Research Council, Cape Town, South Africa; e Faculty of Health Science, McMaster University, Hamilton, Ontario, Canada; f Vagelos College of Physicians and Surgeons, Columbia University, New York, NY, USA; g Department of Global HIV, Hepatitis and Sexually Transmitted Infections Programmes, World Health Organization, Geneva, Switzerland; h Centre for Integrated Data and Epidemiological Research, School of Public Health and Family Medicine, University of Cape Town, Cape Town, South Africa; i Blizard Institute, Faculty of Medicine and Dentistry, Queen Mary University of London, London, UK; j Division of Infectious Diseases and HIV Medicine, Department of Medicine, University of Cape Town, Observatory, Cape Town, South Africa; k Department of Health Research Methods, Evidence, and Impact; l Department of Medicine, McMaster University, Hamilton, Ontario, Canada; m Institute for Infection and Immunity, City St George's, University of London, London, UK; n Wellcome Discovery Research Platforms in Infection, Centre for Infectious Diseases Research in Africa, Institute of Infectious Disease and Molecular Medicine, University of Cape Town, Observatory, Cape Town, South Africa.

**Keywords:** acquired immunodeficiency syndrome, Africa South of the Sahara, CD4-positive T-lymphocytes, HIV, mortality

## Abstract

**Background:**

In sub-Saharan Africa (SSA), people with HIV continue to present with advanced HIV disease (AHD), putting them at high risk of life-threatening opportunistic diseases. We aimed to estimate mortality among this population.

**Methods:**

We conducted a systematic review and meta-analysis of studies reporting one-year mortality among adults living with HIV and presenting to care with CD4^+^ cell count ≤200 cells/mm^3^ in SSA. MEDLINE, EMBASE, and the Cochrane Central Register of Controlled Trials were searched for studies (comprising >500 participants) published between January 1, 2016 and March 21, 2025. Screening and data extraction were done in duplicate. Pooled mortality proportions across CD4^+^ cell count and time strata were calculated using a generalized linear mixed model. Risk of bias was assessed using a modified Newcastle–Ottawa scale. The protocol is registered with PROSPERO, CRD42023451498.

**Results:**

Thirty-six studies with 313 362 participants were included. The weighted median age was 35 years, 64% were female, and 98.9% were antiretroviral therapy-naive. One-year mortality was 12% (95% CI 8–16) among people with CD4^+^ cell count ≤200 cells/mm^3^ and increased with lower CD4^+^ cell count (≤100 cells/mm^3^, 15% (95% CI 11–19); ≤50 cells/mm^3^, 20% (95% CI 12–31)). Most deaths occurred within the first 3 months after AHD presentation. Heterogeneity was substantial. Risk of bias was high in 18 (50%) of 36 included studies.

**Discussion:**

There is high 1-year mortality among people presenting with AHD in SSA. It is a priority to identify AHD with CD4^+^ testing, improve retention in care, and evaluate additional interventions to reduce mortality in this population.

## Introduction

The global decline in HIV-related mortality has plateaued despite expanded access to antiretroviral therapy (ART) and implementation of evidence-based prevention of opportunistic infections. In 2023, approximately 630 000 people died from AIDS-related illnesses, with eastern and southern Africa disproportionately affected [1]. Advanced HIV disease (AHD), defined in adults as a CD4^+^ cell count ≤200 cells/mm^3^ or the presence of a WHO stage 3 or 4 clinical event, is the major contributor to this public health concern [2–6].

Recent household surveys found that approximately 10% of people living with HIV in sub-Saharan Africa have AHD, translating to an estimated 1.88 million individuals (uncertainty interval 1.58–2.20) affected by AHD in the region [7]. This persistent burden of advanced disease is driven by late presentation and, increasingly, by disengagement from care. Illustrating this, approximately two-thirds of people starting ART in the Western Cape province of South Africa are treatment experienced and in several cohort studies AHD is present in up to 30% of people initiating or re-initiating ART [3–6,8–11]. Due to profound immunocompromise, individuals with AHD are at high risk of developing life-threatening opportunistic infections and other HIV-related illnesses, which are the leading reported causes of hospitalization and death in this population [12].

Most of the current knowledge on AHD-associated mortality comes from individual clinical trials or single-center cohort studies. While valuable, these sources provide limited insights around outcomes from broader programmatic settings across sub-Saharan Africa, where variable access to diagnostics, prophylaxis, and treatment may affect HIV outcomes. To better quantify the impact of AHD – defined in this study as people with CD4^+^ cell count ≤200 cells/mm^3^ – and support evidence-based policy, resource allocation, and research prioritization, population-level mortality estimates are needed. We conducted a systematic review and meta-analysis to estimate prevalence of 1-year mortality among adults with CD4^+^ cell count ≤200 cells/mm^3^ in sub-Saharan Africa irrespective of ART status.

## Methods

### Search strategy and selection criteria

For this systematic review and meta-analysis, we searched three electronic databases, including MEDLINE, EMBASE, and the Cochrane Central Register of Controlled Trials (CENTRAL) for studies reporting mortality in people with AHD published between January 1, 2016 and July 14, 2023. This time frame was chosen to capture mortality outcomes in the era of universal test and treat for HIV, following its global adoption in 2016. The search was updated on March 21, 2025. Search terms included combinations of ‘HIV’, ‘mortality’, ‘sub-Saharan Africa’ and ‘adults’ and the search was restricted to articles in English or French. The full search strategies for each database are provided in the appendix (p. 14). Conference abstracts, letters to the editor, guidelines, case reports preprints, and case series were not included.

Studies were eligible if they included at least 500 adults (aged 18 years or older) from sub-Saharan Africa living with HIV and reported mortality at any timepoint within 1 year of identifying a CD4^+^ cell count of ≤200 cells/mm^3^. For studies that reported outcomes on individuals aged <18 or with CD4^+^ cell count >200 cells/mm^3^, we required that at least 90% were adults or at least 90% had CD4^+^ cell count ≤200 cells/mm^3^. If a study did not report the number of participants in each eligibility category, the mean and standard deviation (SD) were used to estimate if 90% of individuals were adults or had CD4^+^ cell count ≤200 cells/mm^3^. If only the median was reported, the mean was assumed to be equal to the median and SD was calculated from the interquartile range (IQR) [13]. Where multiple publications reported on the same cohort of participants, we included the main report or the publication contributing the largest sample size. References were imported into Covidence (Melbourne, Australia). Screening for eligibility (T.C.S., K.D.G., A.C., A.B., S.F., J.L., S.E.) and data extraction were conducted independently in duplicate (T.S. and K.D.G.), with disagreements resolved through discussion, or where necessary, by a third investigator (J.W.E.).

### Data analysis

Summary-level data were extracted directly into a spreadsheet and included: author, year of publication, study period, baseline characteristics of study population (age, sex, CD4^+^ cell count, ART status), trial setting (country, multicenter/single center, hospital/clinic), number of participants with CD4^+^ cell count ≤200 cells/mm^3^, ≤100 cells/mm^3^, and ≤50 cells/mm^3^, number of deaths, timing of deaths, and whether the study was conducted in a specific disease context (e.g., tuberculosis). Countries were grouped into regions as per the United Nations Statistics Division [14].

The primary outcome was mortality at 1 year after study enrolment. If a study reported CD4^+^ cell count within nonstandard thresholds (e.g. ≤150 cells/mm^3^), the population was classified under the next higher standard category (e.g. ≤200 cells/mm^3^). Where no cut off was reported, data were analyzed in the stratum estimated to include 90% of CD4^+^ cell count by using either mean and SD or median and IQR as described above. Baseline characteristics were extracted from the overall study population if not specified for individual CD4 strata. Mortality was extracted for different time points (1 month, 3 months, 6 months, 1 year). If the number of deaths was not reported, mortality was estimated from other sources (e.g., hazard ratios). The IPDfromKM package for R software version 4.4.0. was used to extract mortality data from Kaplan–Meier curves [15,16].

Prespecified subgroup analyses included enrolment period (before vs. after 2016), study design (randomised controlled trial vs. nonrandomised studies), geographic region (Western, Central, Eastern, and Southern Africa) and care setting (clinic vs. hospital). Studies enrolling participants both before and after 2016 were categorised based on the period during which the majority of enrollment occurred. Additional preplanned subgroup analyses included receipt of AHD management components (e.g., co-trimoxazole) and current ART status (naïve vs. experienced), but these were not performed because of limited data.

Risk of bias was assessed independently by two authors (T.S. and K.D.G.) using a modified Newcastle–Ottawa scale (appendix, p. 20). Data from randomised trials were treated as observational, pooling participants irrespective of allocated treatment. Disagreements were resolved by discussion, or if unresolved, by a third reviewer. Risk of bias was classified as low (8–9 points), moderate (6–7 points), or high (<5 points). We assessed the certainty of evidence for all outcomes using the Grading of Recommendations Assessment, Development and Evaluation (GRADE) methodology [17]. The following domains were assessed: precision, consistency, risk of bias, and directness [18–21]. We presented the evidence in a summaries of findings table [22].

Data pooling and statistical analyses were performed using the *metanalysis for single proportion* function and the generalized linear mixed model (GLMM) in the *meta package* in R software version 4.4.0. [16,23]. Summary estimates are presented as proportions with 95% confidence intervals (CIs) calculated using Clopper–Pearson method. Heterogeneity was measured using *I*^2^. Tests for subgroup differences were performed using the *Q*-statistic, comparing pooled mortality estimates across subgroups. Post hoc leave-one-out sensitivity analysis was conducted to assess single-study influence using the random-effects GLMM for proportions (*meta package*).

We followed the PRISMA checklist for reporting this systematic review (appendix, p. 7) [24]. The protocol was registered on PROSPERO (CRD42023451498).

### Role of the funding source

There was no funding source for this study.

## Results

**Fig. 1:**
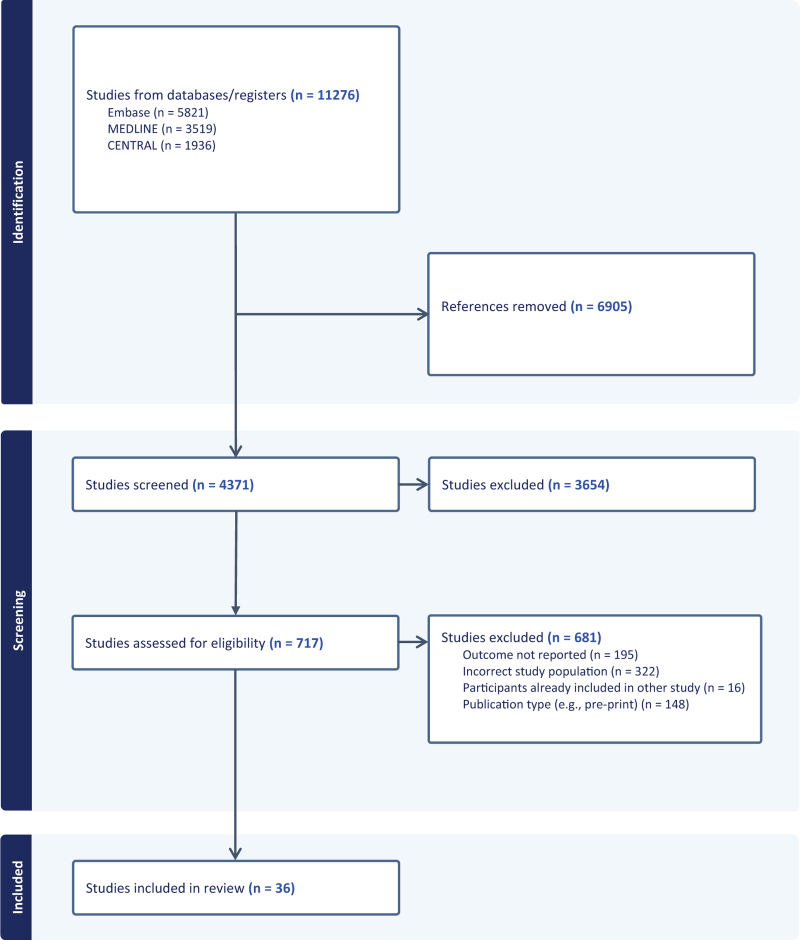
PRISMA flowchart.

Our search identified 11 276 publications, of which 4371 were screened after removing duplicates. Following full-text review of 717 studies, 36 met the eligibility criteria (Fig. 1) [25–60]. The most common reasons for exclusion were sample size <500 or the absence of mortality data within 1 year of identifying a CD4^+^ cell count ≤200 cells/mm^3^. Included studies were conducted in eastern (*n* = 15), southern (*n* = 13), or western Africa (*n* = 1) or across multiple regions (*n* = 7). Study sizes ranged from 541 to 186 863 individuals with CD4^+^ cell count ≤200 cells/mm^3^. Ten studies were randomized controlled trials. Two studies enrolled participants exclusively after 2016. Mortality was most frequently reported at the 1-year timepoint (23 studies). Mortality outcomes for individuals with CD4^+^ cell count of ≤200 cells/mm^3^, ≤100 cells/mm^3^, and ≤50 cells/mm^3^ were reported in 24, 21, and 9 studies, respectively (Table 1).

**Table 1:** Characteristics of included studies.

Author	Country	Design	Setting	Study period	Age, median	Female (%)	≤200	≤100	Median CD4^+^ cell count (IQR)	ART naïve^a^ (%)	Specific population
Amour (2022) [23]	Tanzania	Observational	Multicenter: clinics	01/2015–12/2019	22	87	541		n.r.	n.r.	
Auld (2020) [24]^b^	Botswana	Randomized controlled trial	Multicenter: hospitals and healthcare facilities	08/2012–03/2014	I: 35, II: 34, III: 34	64	7158	3204	I: 184 (100–241), II: 246 (148–310), III: 241 (132–321)	n.r	Tuberculosis
Bassett (2017) [25]	South Africa	Observational	Multicenter: outpatients	08/2010–01/2013	33	49	848		192 (72–346)	100	
Blanc (2020) [26]^b^	Uganda, Ivory Coast, Cambodia, Vietnam	Randomized controlled trial	multicenter: ambulatory	09/2014–05/2017	35	42		583	I: 28 (12–56), II: 32 (13–55)	100	Tuberculosis
Butler (2018) [27]^b^	South Africa	Observational	Single center	01/2004–10/2011	I: 33 II:54	63		6417	n.r.	100	
Chaisson (2019) [28]	Uganda	Observational	Multicenter: clinics	07/2013–12/2016	33	56	711		181 (82–278)	100	
Chimbetete (2020) [29]	Zimbabwe	Observational	Single center: clinic	02/2004–12/2017	33	62		1341	190 (89–314)	100	
Cornell (2017) [30]^b^	South Africa	Observational	Multicentre: clinics and hospitals	2004–2015	I: 38, II: 33	67	43662		148 (71–227)	100	
Drain (2021) [31]	South Africa	Observational	single center: clinic	09/2013–02/2019	33	43	908		107 (52–153)	n.r.	Cryptococcal disease
Faini (2019) [32]	Tanzania	Observational	Single center: clinic	10/2013–07/2015	38	56	<150: 560		61 (26–103)	100	Cryptococcal disease
Fekade (2017) [33]	Ethiopia	Observational	Multicenter: hospitals	01/2009–07/2013	33	61	639	330	144 (78–205)	100	
Grant (2020) [34]	South Africa	Randomized controlled trial	Multicenter: primary healthcare centers	12/2012–12/2014	37	56	<150: 3022		72 (35–112)	n.r.	Tuberculosis
Gupta-Wright (2018) [35]	Malawi, South Africa	Randomized controlled trial	Multicenter: inpatients	10/2015–09/2017	40	57		748	227 (79–436)	8	Tuberculosis
Hakim (2017) [36]	Uganda, Zimbabwe, Malawi, Kenya	Randomized controlled trial	Multicenter	06/2013–04/2015	36	47		1805	37 (16–63)	100	
Hirasen (2018) [37]	South Africa	Observational	Single center; clinics	09/2011–08/2012 and 09/2013–08/2014	38	59	1513	844	171 (70–273)	100	
Honge (2016) [38]	Guinea-Bissau	Observational	Single center: ART clinic	06/2005–12/2014	36	67	1811		206 (89–381)	n.r.	
Hurt (2021) [39]	Botswana	Observational	Multicenter: clinics and hospital	01/2014–01/2016	37	50		1645	54 (25–78)	55	Cryptococcal disease
Inzaule (2022) [40]	Kenya, South Africa, Zambia, Zimbabwe, Uganda	Observational	Multicenter	2007 - 2015	37	58	1972		135 (63–205)	n.r.	
Jarvis (2022) [41]^b^	Botswana, Malawi, South Africa, Uganda	Randomized controlled trial	Multicenter: hospitals	01/2018–02/2021	37	40		814	I: 26 (9–56), II: 28 (11–59)	36	Cryptococcal disease
Kimaro (2019) [42]	Tanzania, Zambia	Randomized controlled trial	Clinic	02/2012–09/2013	n.r.	n.r.	1999	1431	n.r.	n.r.	Cryptococcal disease
Kiragga (2016) [43]^b^	Uganda	Observational	Multicenter: clinics	07/2011–12/2011 and 07/2012–12/2014	32	55		851	I: 34 (12–63), II: 42 (17–71)	100	
Lafort (2018) [44]	Mozambique	Observational	Multicenter: facilities	01/2013–06/2014	n.r.	72		3869	n.r.	n.r.	
Longley (2016) [45]	South Africa	Observational	Multicenter: clinics	05/2011–04/2014	36	53		645	56 (28–78)	100	Cryptococcal disease
Makadzange (2021) [46]	Zimbabwe	Observational	Multicenter: outpatient facilities	04/2015–06/2016	37	44		1320	31 (14–55)	90	Cryptococcal disease
Mody (2020) [47]	Zambia	Observational	Multicenter: clinics	04/2014–07/2015	34	58	9234		268 (134–430)	100	
Moyo (2016) [48]^b^	South Africa	Observational	Multicenter: clinic	07/2007–12/2012	37	59	8263	4050	I: 159 II: 113	100	
Nacarapa (2021) [49]	Mozambique	Observational	Single center: HIV clinic	01/2002–12/2019	35	59	8375		I: 192 (n.r.)	95	
Peter (2016) [50]	South Africa, Tanzania, Zambia, Zimbabwe	Randomized controlled trial	Multicenter: hospitals	01/2013–10/2014	37	51	1725	1272	84 (26–208)	n.r.	Tuberculosis
Sossen (2020) [51]^b^	South Africa	Observational	Multicenter: district hospitals	06/2012–10/2013 and 01/2014–10/2016	36	55	746		I: 150 (56–311), II: 63 (24–131)	40	Tuberculosis
Ssempijja (2020) [52]	Uganda	Observational	Single center	2006 - 2016	34	69		1893	n.r.	n.r.	
Stadelman (2021) [53]^b^	Uganda, South Africa	Includes randomized controlled trial and observational data	Multicenter	I: 11/2010–04/2012, II: 05/2012–06/2013, III: 08/2013–08/2014	I: 36, II: 32	41		977	I: 14 (6, 44), II: 24 (8, 61)	n.r.	Cryptococcal disease
Steytler (2017) [54]^b^	South Africa	Randomized controlled trial	Multicenter: sites	02/2004–12/2007	I: 36, II: 35, III: 36, IV: 35, V: 36, VI: 41	32	1771		I: 99 (40–156), II: 119 (51–175), III: 85 (28–141), IV: 104 (43–153), V: 110 (54–160), VI: 120 (38–156)	n.r.	
Sudfeld (2020) [55]	Tanzania	Randomized controlled trial	Multicenter	02/2014–02/2017	I: 39, II: 39	68	1711		n.r.	n.r.	
Teasdale (2018) [56]	Ethiopia, Kenya, Mozambique, Tanzania	Observational	Multicenter: health facilities	01/2005–12/2014	35	64	186863	95075	164 (78–255)	100	
Tenforde (2019) [57]	Botswana	Observational	Multicenter; hospitals	01/2004–12/2015	37	50	1018	630	139 (63–271)	n.r.	
Worodria (2018) [58]	Uganda	Observational	Single center	04/2011–09/2015	34	48	854		81 (21–226)	n.r.	

Study characteristics						CD4^+^ cell count (cells/mm^3^)					
Author	Country	Cohort/Study name	Design	Setting	Study period	Age, median	Female (%)	≤200	≤100	Median CD4^+^ cell count (IQR)	ART naïve^a^ (%)
Amour (2022) [23]	Tanzania		Observational	Multicenter: clinics	01/2015–12/2019	22	87	541		n.r.	n.r.
Auld (2020) [24]^b^	Botswana	XPRES	Randomized controlled trial	Multicenter: hospitals and healthcare facilities	08/2012–03/2014	I: 35, II: 34, III: 34	64	7158	3204	I: 184 (100–241), II: 246 (148–310), III: 241 (132–321)	n.r
Bassett (2017) [25]	South Africa	Sizanani	Observational	Multicenter: outpatients	08/2010–01/2013	33	49	848		192 (72–346)	100
Blanc (2020) [26]^b^	Uganda, Ivory Coast, Cambodia, Vietnam	STATIS	Randomized controlled trial	multicenter: ambulatory	09/2014–05/2017	35	42		583	I: 28 (12–56), II: 32 (13–55)	100
Butler (2018) [27]^b^	South Africa		Observational	Single center	01/2004–10/2011	I: 33 II:54	63		6417	n.r.	100
Chaisson (2019) [28]	Uganda		Observational	Multicenter: clinics	07/2013–12/2016	33	56	711		181 (82–278)	100
Chimbetete (2020) [29]	Zimbabwe		Observational	Single center: clinic	02/2004–12/2017	33	62		1341	190 (89–314)	100
Cornell (2017) [30]^b^	South Africa	IeDEA-SA	Observational	Multicentre: clinics and hospitals	2004–2015	I: 38, II: 33	67	43662		148 (71–227)	100
Drain (2021) [31]	South Africa		Observational	single center: clinic	09/2013–02/2019	33	43	908		107 (52–153)	n.r.
Faini (2019) [32]	Tanzania	KIULARCO	Observational	Single center: clinic	10/2013–07/2015	38	56	<150: 560		61 (26–103)	100
Fekade (2017) [33]	Ethiopia		Observational	Multicenter: hospitals	01/2009–07/2013	33	61	639	330	144 (78–205)	100
Grant (2020) [34]	South Africa	TB Fast Track	Randomized controlled trial	Multicenter: primary healthcare centers	12/2012–12/2014	37	56	<150: 3022		72 (35–112)	n.r.
Gupta-Wright (2018) [35]	Malawi, South Africa	STAMP	Randomized controlled trial	Multicenter: inpatients	10/2015–09/2017	40	57		748	227 (79–436)	8
Hakim (2017) [36]	Uganda, Zimbabwe, Malawi, Kenya	REALITY	Randomized controlled trial	Multicenter	06/2013–04/2015	36	47		1805	37 (16–63)	100
Hirasen (2018) [37]	South Africa		Observational	Single center; clinics	09/2011–08/2012 and 09/2013–08/2014	38	59	1513	844	171 (70–273)	100
Honge (2016) [38]	Guinea-Bissau	Bissau HIV Cohort	Observational	Single center: ART clinic	06/2005–12/2014	36	67	1811		206 (89–381)	n.r.
Hurt (2021) [39]	Botswana		Observational	Multicenter: clinics and hospital	01/2014–01/2016	37	50		1645	54 (25–78)	55
Inzaule (2022) [40]	Kenya, South Africa, Zambia, Zimbabwe, Uganda	PASER-M	Observational	Multicenter	2007 - 2015	37	58	1972		135 (63–205)	n.r.
Jarvis (2022) [41]^b^	Botswana, Malawi, South Africa, Uganda	AMBITION-cm	Randomized controlled trial	Multicenter: hospitals	01/2018–02/2021	37	40		814	I: 26 (9–56), II: 28 (11–59)	36
Kimaro (2019) [42]	Tanzania, Zambia	REMSTART	Randomized controlled trial	Clinic	02/2012–09/2013	n.r.	n.r.	1999	1431	n.r.	n.r.
Kiragga (2016) [43]^b^	Uganda	ORCAS	Observational	Multicenter: clinics	07/2011–12/2011 and 07/2012–12/2014	32	55		851	I: 34 (12–63), II: 42 (17–71)	100
Lafort (2018) [44]	Mozambique		Observational	Multicenter: facilities	01/2013–06/2014	n.r.	72		3869	n.r.	n.r.
Longley (2016) [45]	South Africa		Observational	Multicenter: clinics	05/2011–04/2014	36	53		645	56 (28–78)	100
Makadzange (2021) [46]	Zimbabwe		Observational	Multicenter: outpatient facilities	04/2015–06/2016	37	44		1320	31 (14–55)	90
Mody (2020) [47]	Zambia		Observational	Multicenter: clinics	04/2014–07/2015	34	58	9234		268 (134–430)	100
Moyo (2016) [48]^b^	South Africa		Observational	Multicenter: clinic	07/2007–12/2012	37	59	8263	4050	I: 159 II: 113	100
Nacarapa (2021) [49]	Mozambique		Observational	Single center: HIV clinic	01/2002–12/2019	35	59	8375		I: 192 (n.r.)	95
Peter (2016) [50]	South Africa, Tanzania, Zambia, Zimbabwe		Randomized controlled trial	Multicenter: hospitals	01/2013–10/2014	37	51	1725	1272	84 (26–208)	n.r.
Sossen (2020) [51]^b^	South Africa	SILVAMP TB LAM	Observational	Multicenter: district hospitals	06/2012–10/2013 and 01/2014–10/2016	36	55	746		I: 150 (56–311), II: 63 (24–131)	40
Ssempijja (2020) [52]	Uganda	MHCHC	Observational	Single center	2006 - 2016	34	69		1893	n.r.	n.r.
Stadelman (2021) [53]^b^	Uganda, South Africa	ASTRO CM and others	Includes randomized controlled trial and observational data	Multicenter	I: 11/2010–04/2012, II: 05/2012–06/2013, III: 08/2013–08/2014	I: 36, II: 32	41		977	I: 14 (6, 44), II: 24 (8, 61)	n.r.
Steytler (2017) [54]^b^	South Africa	Phidisa-II	Randomized controlled trial	Multicenter: sites	02/2004–12/2007	I: 36, II: 35, III: 36, IV: 35, V: 36, VI: 41	32	1771		I: 99 (40–156), II: 119 (51–175), III: 85 (28–141), IV: 104 (43–153), V: 110 (54–160), VI: 120 (38–156)	n.r.
Sudfeld (2020) [55]	Tanzania	ToV-4	Randomized controlled trial	Multicenter	02/2014–02/2017	I: 39, II: 39	68	1711		n.r.	n.r.
Teasdale (2018) [56]	Ethiopia, Kenya, Mozambique, Tanzania		Observational	Multicenter: health facilities	01/2005–12/2014	35	64	186863	95075	164 (78–255)	100
Tenforde (2019) [57]	Botswana	Botswana national meningitis survey	Observational	Multicenter; hospitals	01/2004–12/2015	37	50	1018	630	139 (63–271)	n.r.
Worodria (2018) [58]	Uganda	MIND-IHOP	Observational	Single center	04/2011–09/2015	34	48	854		81 (21–226)	n.r.

n.r., not reported.

a Either as reported by the authors or as people without previous ART exposure.

b Roman numerals reflect baseline characteristics of different study arms or periods.

Outcomes were reported for 313 362 unique participants, including 228 296 with a CD4^+^ cell count ≤200 cells/mm^3^, 126 910 with a CD4^+^ cell count ≤100 cells/mm^3^, and 59 189 with a CD4^+^ cell count ≤50 cells/mm^3^. The weighted median age was 35 years (range of medians: 22–52 years) and 200 552 (64%) were female. Where reported, the weighted proportion of ART-naïve participants was 98.9% (range: 8–100%).

**Fig. 2:**
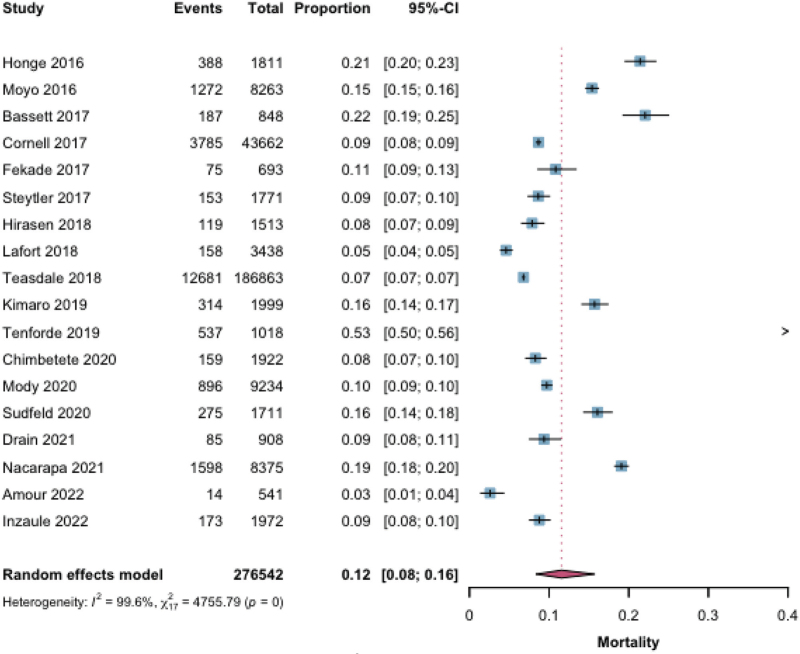
One-year mortality for CD4^+^ cell ≤200 cells/mm^3^.

**Fig. 3:**
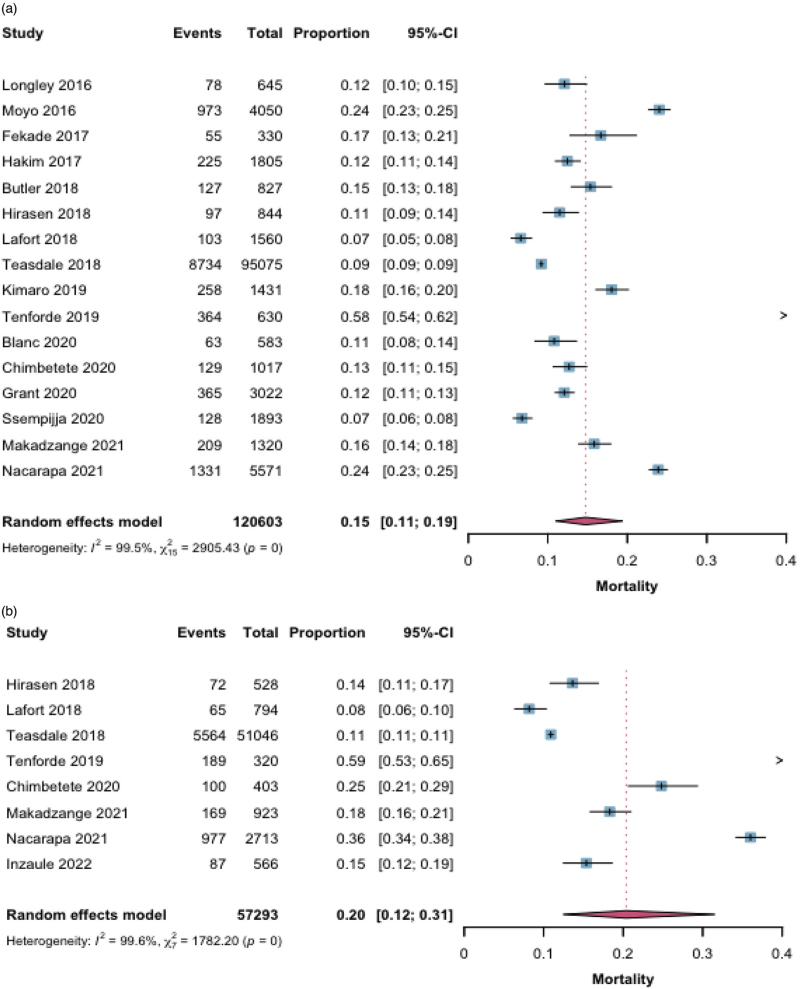
One-year mortality for (a) CD4^+^ cell count ≤100 cells/mm^3^ and (b) CD4^+^ cell count ≤50 cells/mm^3^.

One-year mortality after identification of a CD4^+^ cell count ≤200 cells/mm^3^ was reported for 276 542 individuals across 18 studies. The pooled mortality estimate was 12% (95% CI 8–16) (Fig. 2). For 1-year mortality among individuals with CD4^+^ cell count ≤200 cells/mm^3^, evidence from nonrandomized studies (270 368 participants, 14 studies) suggested a pooled mortality of 11% (95% CI 7–17), but the certainty of evidence was rated low, mainly due to considerable heterogeneity (*I*^2^ ≥ 99.7%), moderate–high risk of bias, and indirectness. Evidence from randomized controlled trials (6174 participants, 4 studies) suggested a mortality of 13% (95% CI 10–16), with certainty rated very low because of high heterogeneity (*I*^2^ ≥ 94.8%) and indirectness, despite generally lower risk of bias. For individuals with CD4^+^ cell count ≤100 cells/mm^3^, pooled 1-year mortality was 15% (95% CI 11–19) based on 120 603 participants from 16 studies, with certainty of evidence rated low due to heterogeneity, risk of bias, and indirectness. Among those with CD4^+^ cell count ≤50 cells/mm^3^, mortality was higher at 20% (95% CI 12–31%) (57 293 participants, 8 studies), but the certainty of evidence was very low due to very high heterogeneity, small number of studies, and imprecision (Fig. 3, Table 2).

Blue squares show point estimates (vertical black line) and horizontal black line represents 95% CI. Red diamond represents pooled estimate including 95% CI. Dotted red line shows the overall pooled estimate.

Blue squares show point estimates (vertical black line) and horizontal black line represents 95% CI. Red diamond represents pooled estimate including 95% CI. Dotted red line shows the overall pooled estimate.

**Table 2:**
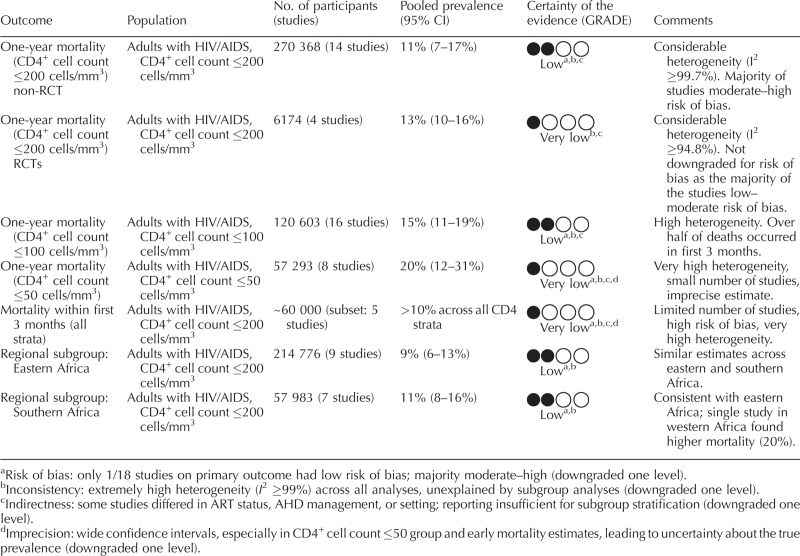
Summary of findings.

**Fig. 4:**
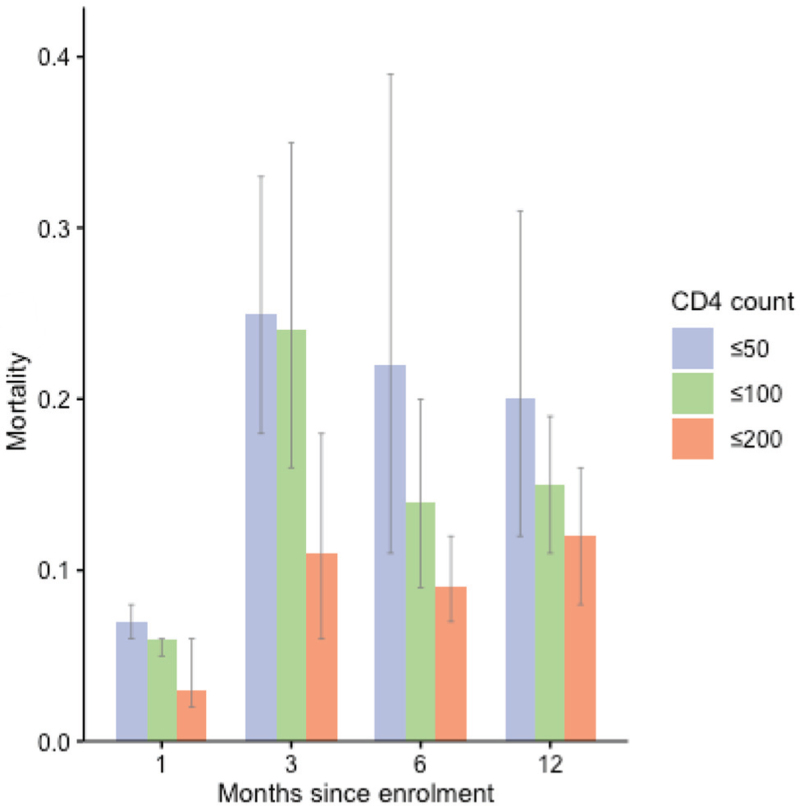
Mortality at discrete timepoints in the first year stratified by CD4^+^ cell count.

**Fig. 5:**
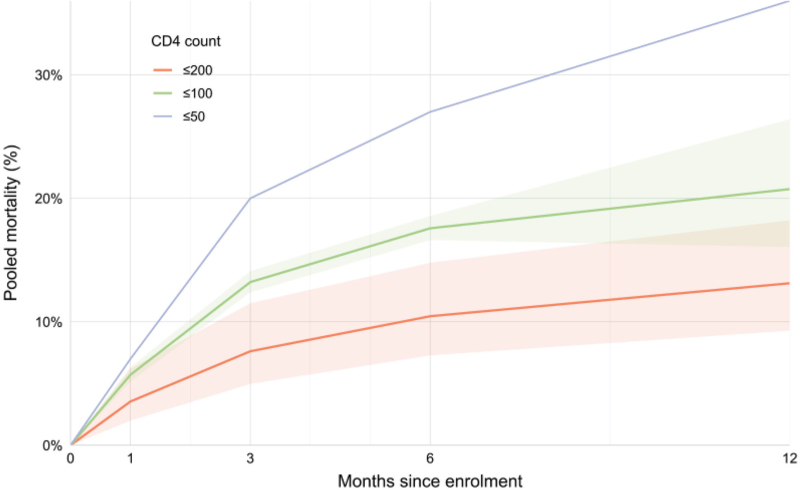
Pooled mortality by time since enrolment, stratified by CD4^+^ cell count.

Across all CD4 strata, early mortality within the first 3 months exceeded 10% (>60 000 participants, 5 studies). The certainty of evidence was very low, reflecting sparse data, high risk of bias, and very high heterogeneity (Fig. 4, Table 2). In the sub-set of studies that reported mortality at all timepoints (1, 3, 6, and 12 months), a similar early concentration of deaths was observed (Fig. 5), with more than half of all deaths occurring within the first 3 months (appendix, p. 6). Leave-one-out sensitivity analyses yielded pooled mortality between 10 and 12% and did not alter overall conclusions (appendix, p. 5).

Bars indicate pooled estimate and lines represent 95% CI.

Only studies reporting mortality at all specified timepoints were included. Number of studies: CD4^+^ cell count ≤200 cells/mm^3^: 5; CD4^+^ cell count ≤100 cells/mm^3^: 2; CD4^+^ cell count ≤50 cells/mm^3^: 1. Shaded areas represent 95% confidence intervals (not included for the CD4^+^ cell count <50 stratum as this was based on a single study).

Pooled mortality was higher in studies enrolling participants before 2016 (12% [95% CI 8–16], 16 studies, 276 542 participants) than in studies enrolling after 2016 (5% [95% CI 2–12], 2 studies, 1449 participants) (*P*-value for subgroup differences = 0.055) (appendix p. 1). Subgroup analysis by study design showed similar mortality estimates in nonrandomised studies (11% [95%CI 7–17], 14 studies, 270 368 participants) and randomised trials (13% [95%CI 10–16], 4 studies, 6174 participants) (*P*-value for subgroup differences = 0.67) (appendix, p. 2). Pooled mortality estimates were similar from studies in eastern (9 studies, 214 776 participants) and southern Africa (7 studies, 57 983 participants) with a mortality of 9% (95% CI 6–13) and 11% (95% CI 8–16) (*P*-value for subgroup differences = 0.17), respectively (appendix, p. 3). Data from other regions could not be pooled due to a limited number of studies. The only study conducted exclusively in western Africa (1811 participants) reported mortality of 20% (95% CI 18–22). In subgroups defined by care setting, pooled mortality was 11% (95% CI 8–16) in clinic-based cohorts (11 studies, 37 185 participants) and 27% (95% CI 7–63) in hospital-based cohorts (2 studies, 1111 participants) without statistical evidence of heterogeneity (p-value for subgroup differences = 0.193) (appendix, p. 4). Subgroup analyses by AHD management components and ART status could not be conducted due to insufficient reporting across studies.

Risk of bias was low in 2 (6%), moderate in 16 (44%), and high in 18 (50%) of the 36 included studies. Among the 18 studies reporting on the primary outcome (mortality at one year among individuals with a CD4^+^ cell count ≤200 cells/mm^3^), risk of bias was assessed as low in 1 study (6%), moderate in 11 studies (61%), and high in 6 studies (33%) (appendix p. 12).

## Discussion

Adults with advanced HIV disease, defined in our analysis as a CD4^+^ cell count ≤200 cells/mm^3^, experience high early mortality with an estimated 12% dying within the first year of entry or re-entry to care, with mortality increasing as CD4^+^ cell count declines. These findings highlight the persistent challenge of AHD in the region.

Most deaths occur within the first 3 months after presentation with a CD4^+^ cell count ≤200 cells/mm^3^, reflecting vulnerability to rapid clinical deterioration, even in the context of ART initiation. This early mortality is likely driven by preventable opportunistic infections – particularly tuberculosis and severe bacterial infection – which remain leading causes of hospitalization and death despite the availability of ART and WHO-recommended AHD care packages [2,61–63]. Interventions such as antigen screening and preemptive therapy for cryptococcal disease and urine lipoarabinomannan testing for tuberculosis have shown promise in clinical trials, but their real-world impact is limited by implementation barriers, including diagnostic stockouts and attrition along the screening cascade [37,64]. The REALITY trial demonstrated that a package of enhanced prophylaxis that included azithromycin reduced mortality among individuals with CD4^+^ cell count ≤100 cells/mm^3^[38]. However, azithromycin has not been adopted into guidelines due to uncertainty about its independent effect. The declining availability of CD4 testing, linked to a programmatic shift towards prioritizing viral load monitoring under the universal test and treat strategy, has further reduced the ability to risk stratify patients and allow timely delivery of guideline-recommended management [65,66]. These observations highlight the need to improve implementation of existing interventions and to develop new strategies to reduce mortality in this population.

Our analysis suggests a trend toward lower mortality in studies conducted after 2016, potentially reflecting advances in HIV care including broader access and more rapid initiation of ART thereby reducing loss to follow-up [1,3,67,68]. However, the observation of lower mortality after 2016 is based on only two relatively small and heterogeneous studies, resulting in considerable uncertainty in the estimate of mortality, as also reflected by the wide confidence intervals (95% CI 0.02; 0.12). The lack of contemporary data underscores the need for improved data collection and reporting to assess the effectiveness of current strategies. Geographic representation was skewed toward eastern and southern Africa, with limited data from western and central regions. This restricts generalizability and further highlights the need for expanded data collection. Notably, the only study from western Africa reported higher mortality, suggesting regional variation in outcomes. Comparing care settings, higher hospital mortality was driven by one cohort undergoing lumbar puncture for suspected meningitis. In leave-one-out sensitivity of the primary analysis, omitting this study had the largest impact, but did not change conclusions. It therefore seems likely that baseline acuity and opportunistic infection burden, rather than enrolment setting, explains the differences. High heterogeneity (*I*^2^ > 99%) was observed across studies and resulted in downgrading of the certainty of evidence (GRADE). While expected in proportional meta-analyses, this likely reflects true clinical and programmatic variation, including differences in ART status, access to diagnostics, and implementation of AHD care [7,13,69–73].

Several limitations warrant consideration. First, our reliance on CD4^+^ cell count to define AHD may exclude individuals with WHO stage 3 or 4 disease, who may have different outcomes. However, concordance between clinical disease stage and CD4^+^ cell count is poor, and a CD4-based approach provides an objective and reproducible definition [74–76]. Second, loss to follow-up was substantial or unreported in many studies and there was heterogeneity in outcome ascertainment, potentially underestimating mortality [77]. Third, included cohorts were predominantly ART-naïve, likely over-representing first-time presenters and under-representing people re-engaging in care with AHD. This limits generalizability of our findings to the broader AHD population where a substantial proportion of people with AHD are treatment experienced [78,79]. However, we cannot exclude the possibility that ART status at baseline was incorrect because of missing information. Fourth, some planned subgroup analyses (e.g. ART status, and receipt of AHD management package components) could not be conducted due to insufficient data. Fifth, our search strategy was limited to English and French language publications and excluded grey literature. Finally, we acknowledge that RCTs are not designed to estimate prevalence and often include selective populations, limiting generalizability to the wider public. This may introduce selection bias and reduce the applicability of findings for public health planning. However, a sub-group analysis showed similar mortality estimates for RCTs and non-RCTs.

In conclusion, mortality among individuals presenting with AHD in sub-Saharan Africa remains unacceptably high, particularly in the early months of care. National HIV programs must prioritize AHD by restoring CD4 testing capacity, ensuring consistent implementation of the WHO care package, and improving access to diagnostics and treatments for leading causes of death. Future research should focus on better identifying causes of death, evaluating new interventions, preventing disengagement from care, and include specific patient populations within AHD such as children or adolescents.

## Acknowledgements

T.S., G.M., J.E., and S.W. designed the study. T.S., N.F., D.M., J.E., and S.W. wrote the study protocol. T.C.S., K.D.G., A.C., A.B., S.F., J.L., and S.E. screened the articles and extracted data from the articles included in the meta-analysis. T.S. and K.D.G. supervised screening and data extraction. T.S., K.D.G., and M.E. assessed the risk of bias and quality of studies. T.S., K.D.G., and M.E. accessed and verified the data. T.S. and K.D.G. did the statistical analyses. A.H. and M.E. conducted the GRADE assessment. T.S., K.D.G., and S.W. drafted the first version of the manuscript. S.W., J.W.E., D.M., N.F., M.E., A.H., and G.M. critically reviewed the manuscript. All authors had full access to all the data in the study and had final responsibility for the decision to submit for publication. All authors approved the final manuscript.

We want to thank Jo-Anne Petropoulos for her support in developing the search strategy.

Data sharing: The data from this study will be made available to researchers upon request to the corresponding author.

### Conflicts of interest

There are no conflicts of interest.

## Supplementary Material

Supplemental Digital Content
