# Supplementary material for: One-year mortality among adults with advanced HIV in sub-Saharan Africa: a systematic review and meta-analysis
Source: AIDS. 2026 Jan 22;40(7):967–81. doi: 10.1097/QAD.0000000000004431 (PMC7618665; doi:10.1097/QAD.0000000000004431)
Supplement: Supplemental Digital Content [file aids-40-0967-s001.docx]

Supplementary Figure S1: Mortality at one year for CD4 ≤200cells/mm^3^ subgroup based on year of enrollment


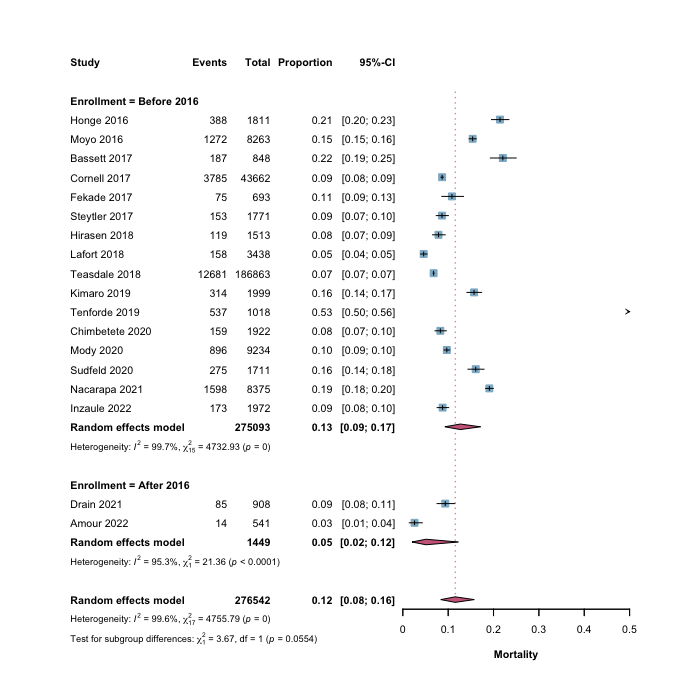


Supplementary Figure S2: Mortality at one year for CD4 ≤200cells/mm^3^ subgroup based on study design


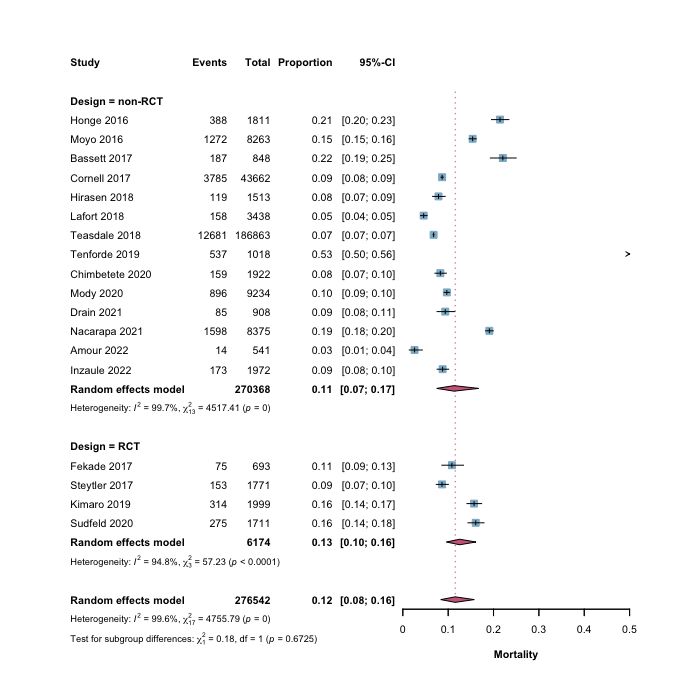


Supplementary Figure S3: Mortality at one year for CD4 ≤200cells/mm^3^ subgroup based on geographical location


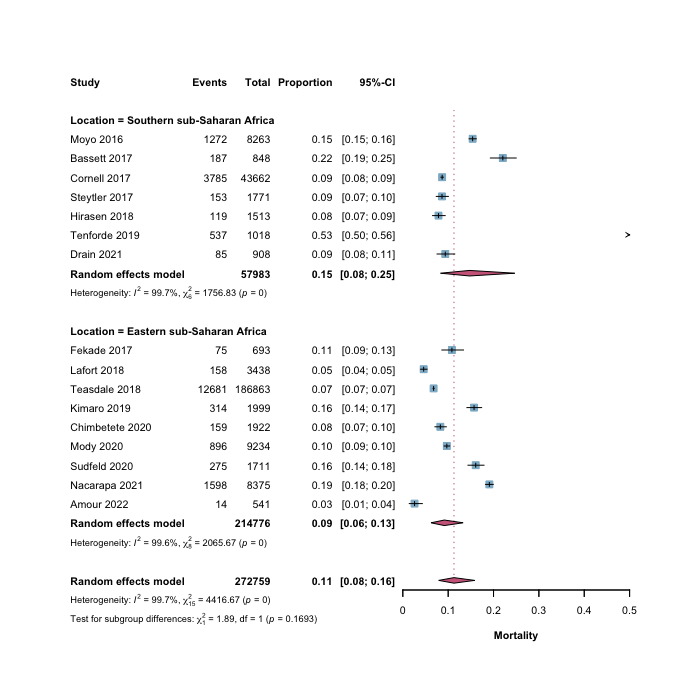


Supplementary Figure S4: Mortality at one year for CD4 ≤200cells/mm^3^ subgroup based on enrolment facility


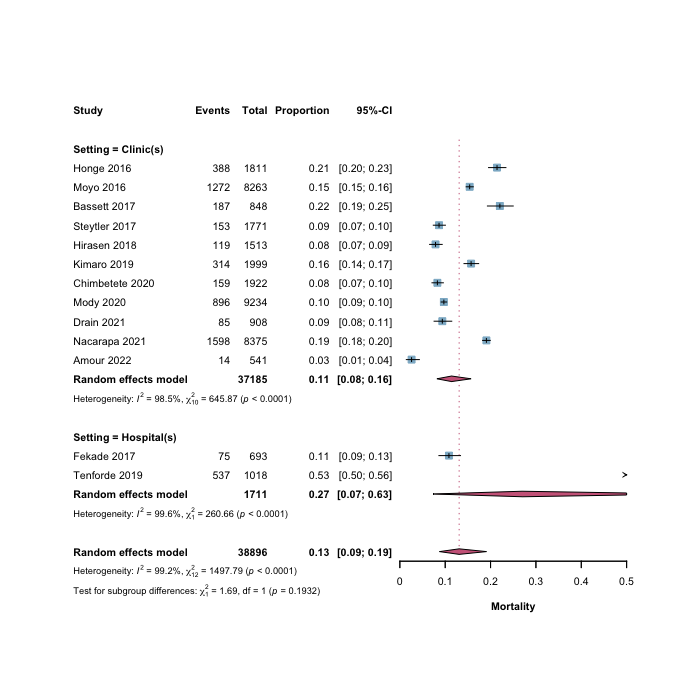


Supplementary Figure S5: Leave-one-out sensitivity analysis of pooled mortality at one year for CD4 ≤200cells/mm^3^


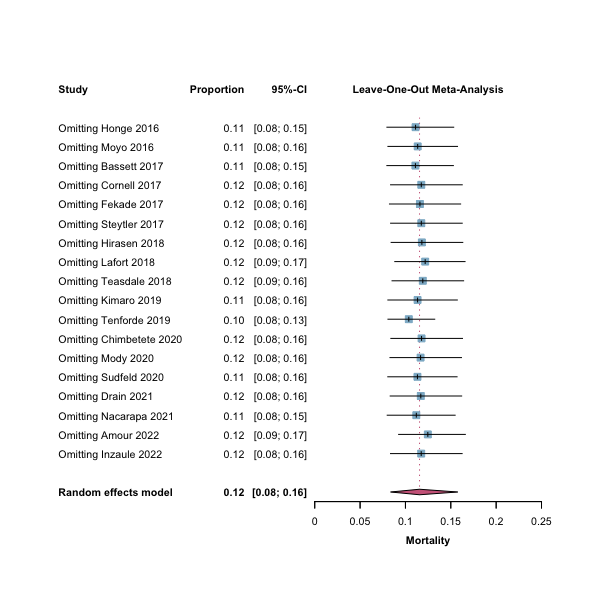
Supplementary Table S1: Cumulative mortality and absolute increase over time, stratified by baseline CD4 count

| CD4 stratum | Time (months) | Cumulative mortality (%) | Absolute increase (%) |
| --- | --- | --- | --- |
| ≤200 cells/μL | 1 | 4.17 | Ref |
|  | 3 | 9.64 | 5.47 |
|  | 6 | 12.95 | 3.31 |
|  | 12 | 16.96 | 4.01 |
| ≤100 cells/μL | 1 | 5.71 | Ref |
|  | 3 | 13.22 | 7.51 |
|  | 6 | 17.57 | 4.36 |
|  | 12 | 23.49 | 5.91 |

**Supplement Table S2: PRISMA checklist**

| **Section and Topic** | **Item #** | **Checklist item** | **Location where item is reported** |
| --- | --- | --- | --- |
| **TITLE** | | |  |
| Title | 1 | Identify the report as a systematic review. | P1 |
| **ABSTRACT** | | |  |
| Abstract | 2 | See the PRISMA 2020 for Abstracts checklist. | Followed, 4-5 |
| **INTRODUCTION** | | |  |
| Rationale | 3 | Describe the rationale for the review in the context of existing knowledge. | Introduction |
| Objectives | 4 | Provide an explicit statement of the objective(s) or question(s) the review addresses. | Introduction |
| **METHODS** | | |  |
| Eligibility criteria | 5 | Specify the inclusion and exclusion criteria for the review and how studies were grouped for the syntheses. | Methods |
| Information sources | 6 | Specify all databases, registers, websites, organisations, reference lists and other sources searched or consulted to identify studies. Specify the date when each source was last searched or consulted. | Methods |
| Search strategy | 7 | Present the full search strategies for all databases, registers and websites, including any filters and limits used. | Overview in Methods, detailed Appendix |
| Selection process | 8 | Specify the methods used to decide whether a study met the inclusion criteria of the review, including how many reviewers screened each record and each report retrieved, whether they worked independently, and if applicable, details of automation tools used in the process. | Methods |
| Data collection process | 9 | Specify the methods used to collect data from reports, including how many reviewers collected data from each report, whether they worked independently, any processes for obtaining or confirming data from study investigators, and if applicable, details of automation tools used in the process. | Methods |
| Data items | 10a | List and define all outcomes for which data were sought. Specify whether all results that were compatible with each outcome domain in each study were sought (e.g. for all measures, time points, analyses), and if not, the methods used to decide which results to collect. | Methods |
|  | 10b | List and define all other variables for which data were sought (e.g. participant and intervention characteristics, funding sources). Describe any assumptions made about any missing or unclear information. | Methods |
| Study risk of bias assessment | 11 | Specify the methods used to assess risk of bias in the included studies, including details of the tool(s) used, how many reviewers assessed each study and whether they worked independently, and if applicable, details of automation tools used in the process. | Methods |
| Effect measures | 12 | Specify for each outcome the effect measure(s) (e.g. risk ratio, mean difference) used in the synthesis or presentation of results. | Methods |
| Synthesis methods | 13a | Describe the processes used to decide which studies were eligible for each synthesis (e.g. tabulating the study intervention characteristics and comparing against the planned groups for each synthesis (item #5)). | Methods |
|  | 13b | Describe any methods required to prepare the data for presentation or synthesis, such as handling of missing summary statistics, or data conversions. | Methods |
|  | 13c | Describe any methods used to tabulate or visually display results of individual studies and syntheses. | Methods |
|  | 13d | Describe any methods used to synthesize results and provide a rationale for the choice(s). If meta-analysis was performed, describe the model(s), method(s) to identify the presence and extent of statistical heterogeneity, and software package(s) used. | Methods |
|  | 13e | Describe any methods used to explore possible causes of heterogeneity among study results (e.g. subgroup analysis, meta-regression). | Methods |
|  | 13f | Describe any sensitivity analyses conducted to assess robustness of the synthesized results. | Methods |
| Reporting bias assessment | 14 | Describe any methods used to assess risk of bias due to missing results in a synthesis (arising from reporting biases). | Methods |
| Certainty assessment | 15 | Describe any methods used to assess certainty (or confidence) in the body of evidence for an outcome. | Methods |
| **RESULTS** | | |  |
| Study selection | 16a | Describe the results of the search and selection process, from the number of records identified in the search to the number of studies included in the review, ideally using a flow diagram. | Results |
|  | 16b | Cite studies that might appear to meet the inclusion criteria, but which were excluded, and explain why they were excluded. | Results |
| Study characteristics | 17 | Cite each included study and present its characteristics. | Table 1 |
| Risk of bias in studies | 18 | Present assessments of risk of bias for each included study. | Reported in Results, Detailed assessment in Appendix |
| Results of individual studies | 19 | For all outcomes, present, for each study: (a) summary statistics for each group (where appropriate) and (b) an effect estimate and its precision (e.g. confidence/credible interval), ideally using structured tables or plots. | Forest plots (Figures 2-5), and Appendix |
| Results of syntheses | 20a | For each synthesis, briefly summarise the characteristics and risk of bias among contributing studies. | Results |
|  | 20b | Present results of all statistical syntheses conducted. If meta-analysis was done, present for each the summary estimate and its precision (e.g. confidence/credible interval) and measures of statistical heterogeneity. If comparing groups, describe the direction of the effect. | In forest plots and Results |
|  | 20c | Present results of all investigations of possible causes of heterogeneity among study results. | Appendix and Results |
|  | 20d | Present results of all sensitivity analyses conducted to assess the robustness of the synthesized results. | Appendix and Results |
| Reporting biases | 21 | Present assessments of risk of bias due to missing results (arising from reporting biases) for each synthesis assessed. | Appendix |
| Certainty of evidence | 22 | Present assessments of certainty (or confidence) in the body of evidence for each outcome assessed. | Table 2 |
| **DISCUSSION** | | |  |
| Discussion | 23a | Provide a general interpretation of the results in the context of other evidence. | Discussion |
|  | 23b | Discuss any limitations of the evidence included in the review. | Discussion |
|  | 23c | Discuss any limitations of the review processes used. | Discussion |
|  | 23d | Discuss implications of the results for practice, policy, and future research. | Discussion |
| **OTHER INFORMATION** | | |  |
| Registration and protocol | 24a | Provide registration information for the review, including register name and registration number, or state that the review was not registered. | Appendix and Methods |
|  | 24b | Indicate where the review protocol can be accessed, or state that a protocol was not prepared. | Methods |
|  | 24c | Describe and explain any amendments to information provided at registration or in the protocol. | Methods |
| Support | 25 | Describe sources of financial or non-financial support for the review, and the role of the funders or sponsors in the review. | Methods |
| Competing interests | 26 | Declare any competing interests of review authors. | Declaration of interest |
| Availability of data, code and other materials | 27 | Report which of the following are publicly available and where they can be found: template data collection forms; data extracted from included studies; data used for all analyses; analytic code; any other materials used in the review. | Data sharing |

*From:*  Page MJ, McKenzie JE, Bossuyt PM, Boutron I, Hoffmann TC, Mulrow CD, et al. The PRISMA 2020 statement: an updated guideline for reporting systematic reviews. BMJ 2021;372:n71. doi: 10.1136/bmj.n71. This work is licensed under CC BY 4.0. To view a copy of this license, visit <https://creativecommons.org/licenses/by/4.0/>

**Supplement Table S3: Risk of bias assessment**

|  |  | **Selection** | | | | **Comparability** | **Outcome** | | |  |
| --- | --- | --- | --- | --- | --- | --- | --- | --- | --- | --- |
| **Author** | **Year** | **Q1** | **Q2** | **Q3** | **Q4** | **Q1** | **Q1** | **Q2** | **Q3** | **Total *** |
| Amour | 2022 | 0 | * | * | * | * | 0 | * | 0 | 5 |
| Auld | 2020 | * | * | * | * | * | * | * | * | 8 |
| Bassett | 2017 | * | * | 0 | * | 0 | * | * | * | 6 |
| Blanc | 2020 | * | * | * | * | * | * | * | 0 | 7 |
| Butler | 2018 | * | 0 | * | * | * | * | * | 0 | 6 |
| Chaisson | 2019 | 0 | * | 0 | * | * | * | * | 0 | 5 |
| Chimbetete | 2020 | * | 0 | * | * | * | * | * | * | 7 |
| Cornell | 2017 | * | 0 | * | * | * | * | * | 0 | 6 |
| Drain | 2021 | * | 0 | * | * | * | * | * | 0 | 6 |
| Faini | 2019 | 0 | * | * | * | 0 | 0 | * | 0 | 4 |
| Fekade | 2017 | * | * | * | * | ** | * | * | 0 | 8 |
| Grant | 2020 | 0 | * | * | * | 0 | * | * | * | 6 |
| Gupta-Wright | 2018 | 0 | * | 0 | * | 0 | * | * | * | 5 |
| Hakim | 2017 | 0 | * | * | * | ** | * | * | 0 | 7 |
| Hirasen | 2018 | 0 | * | * | * | * | * | * | 0 | 6 |
| Honge | 2016 | * | 0 | 0 | * | 0 | 0 | * | 0 | 3 |
| Hurt | 2021 | 0 | * | * | * | 0 | * | * | 0 | 5 |
| Inzaule | 2022 | * | * | * | * | * | * | * | 0 | 7 |
| Jarvis | 2022 | 0 | * | * | 0 | 0 | * | * | * | 5 |
| Kimaro | 2019 | * | * | * | * | * | 0 | * | 0 | 6 |
| Kiragga | 2016 | 0 | 0 | * | * | 0 | 0 | * | 0 | 3 |
| Lafort | 2018 | * | * | 0 | * | * | 0 | * | 0 | 5 |
| Longley | 2016 | 0 | * | 0 | * | 0 | * | * | * | 5 |
| Makadzange | 2021 | 0 | * | * | * | * | * | * | 0 | 6 |
| Mody | 2020 | * | * | * | * | 0 | * | * | 0 | 6 |
| Moyo | 2016 | * | 0 | * | * | * | * | * | 0 | 6 |
| Nacarapa | 2021 | * | 0 | * | * | * | 0 | * | 0 | 5 |
| Peter | 2016 | 0 | * | 0 | * | 0 | * | * | 0 | 4 |
| Sossen | 2020 | 0 | 0 | 0 | * | 0 | * | * | 0 | 3 |
| Ssempijja | 2020 | * | 0 | * | * | * | 0 | * | 0 | 5 |
| Stadelman | 2021 | 0 | 0 | 0 | * | 0 | * | * | * | 4 |
| Steytler | 2017 | * | * | * | * | * | 0 | * | 0 | 6 |
| Sudfeld | 2020 | 0 | * | 0 | * | * | * | * | * | 6 |
| Teasdale | 2018 | * | 0 | 0 | * | * | * | * | 0 | 5 |
| Tenforde | 2019 | 0 | 0 | 0 | * | 0 | * | * | * | 4 |
| Worodria | 2018 | 0 | * | 0 | * | 0 | 0 | * | 0 | 3 |

**Supplement List S1: Search Strategy**

**Central:**

1. exp hiv infections/ or acquired immunodeficiency syndrome / or aids-related complex/ or aids-related opportunistic infections/

2. (advanced HIV disease or hiv infection* or acquired immunodeficiency syndrome or aids-related complex or aids-related opportunistic infections).mp.

3. (acquired immun* and deficiency syndrome).mp.

4. (Hiv or "hiv‐1" or "hiv‐2*" or "hiv1" or "hiv2" or hiv infect*).mp.

5. ("human immunodeficiency virus" or "human immunedeficiency virus" or "human immuno‐deficiency virus" or "human immune‐deficiency virus").mp.

6. ("acquired immunodeficiency syndrome" or "acquired immunedeficiency syndrome" or "acquired immuno‐deficiency syndrome" or "acquired immune‐deficiency syndrome" or (acquired immun* and deficiency syndrome)).mp.

7. aids.mp.

8. or/1-7

9. mortality/ or "cause of death"/ or fatal outcome/ or hospital mortality/ or mortality, premature/ or survival rate/

10. (mortalit* or death* or dying or survival rate*).mp.

11. 9 or 10

12. "sub–Saharan Africa".mp.

13. Subsaharan Africa.mp.

14. exp "Africa South of the Sahara"/

15. (ANGOLA or BENIN or BOTSWANA or BURKINA FASO or CABO VERDE or CAMEROON or CENTRAL AFRICAN REPUBLIC or CHAD or CONGO or COTE D?IVOIRE or DEMOCRATIC REPUBLIC OF THE CONGO or DJIBOUTI or EQUATORIAL GUINEA or ERITREA or ESWATINI or ETHIOPIA or GABON or GAMBIA or GHANA or GUINEA or GUINEA-BISSAU or KENYA or LESOTHO or LIBERIA or MALAWI or MALI or MAURITANIA or MOZAMBIQUE or NAMIBIA or NIGER or NIGERIA or RWANDA or "SAO TOME AND PRINCIPE" or SENEGAL or SIERRA LEONE or SOMALIA or SOUTH AFRICA or SOUTH SUDAN or SUDAN or TANZANIA or TOGO or UGANDA or ZAMBIA or ZIMBABWE).mp. [mp=title, original title, abstract, floating sub-heading word, mesh headings, heading words, keyword]

16. or/12-15

17. 8 and 11 and 16

18. adult/ or aged/ or "aged, 80 and over"/ or centenarians/ or nonagenarians/ or octogenarians/ or frail elderly/ or middle aged/ or young adult/

19. (adult* or aged or centenarian? or nonagenarian? or octogenarian? or elderly or senior?).mp.

20. 18 or 19

21. 17 and 20

22. limit 17 to ("young adult (19 to 24 years)" or "adult (19 to 44 years)" or "young adult and adult (19-24 and 19-44)" or "middle age (45 to 64 years)" or "middle aged (45 plus years)" or "all aged (65 and over)" or "aged (80 and over)")

23. 21 or 22

24. limit 23 to yr="2016 -Current"

25. limit 24 to (english or french)

**Embase**

1 exp human immunodeficiency virus infection/ or acquired immune deficiency syndrome/ or human immunodeficiency virus 1 infection/ or human immunodeficiency virus 2 infection/

2 (advanced HIV disease or hiv infection* or acquired immunodeficiency syndrome or aids-related complex or aids-related opportunistic).mp.

3 (acquired immun* and deficiency syndrome).mp.

4 (Hiv or "hiv‐1" or "hiv‐2*" or "hiv1" or "hiv2" or hiv infect*).mp.

5 ("human immunodeficiency virus" or "human immunedeficiency virus" or "human immuno‐deficiency virus" or "human immune‐deficiency virus").mp.

6 ("acquired immunodeficiency syndrome" or "acquired immunedeficiency syndrome" or "acquired immuno‐deficiency syndrome" or "acquired immune‐deficiency syndrome" or (acquired immun* and deficiency syndrome)).mp.

7 aids.mp.

8 or/1-7

9 excess mortality/ or out-of-hospital mortality/ or in-hospital mortality/ or mortality/ or mortality risk/ or hospital mortality/ or all cause mortality/ or standardized mortality ratio/ or mortality rate/

10 (mortalit* or death* or dying or survival rate*).mp.

11 9 or 10 3440208

12 "sub–Saharan Africa".mp.

13 Subsaharan Africa.mp.

14 exp "Africa south of the Sahara"/

15 (ANGOLA or BENIN or BOTSWANA or BURKINA FASO or CABO VERDE or CAMEROON or CENTRAL AFRICAN REPUBLIC or CHAD or CONGO or COTE D?IVOIRE or DEMOCRATIC REPUBLIC OF THE CONGO or DJIBOUTI or EQUATORIAL GUINEA or ERITREA or ESWATINI or ETHIOPIA or GABON or GAMBIA or GHANA or GUINEA or GUINEA-BISSAU or KENYA or LESOTHO or LIBERIA or MALAWI or MALI or MAURITANIA or MOZAMBIQUE or NAMIBIA or NIGER or NIGERIA or RWANDA or "SAO TOME AND PRINCIPE" or SENEGAL or SIERRA LEONE or SOMALIA or SOUTH AFRICA or SOUTH SUDAN or SUDAN or TANZANIA or TOGO or UGANDA or ZAMBIA or ZIMBABWE).mp.

16 or/12-15

17 exp cohort analysis/

18 exp longitudinal study/

19 exp prospective study/

20 exp follow up/ 2048478

21 cohort$.tw. 1467739

22 exp case control study/

23 (case$ and control$).tw.

24 or/17-23

25 Clinical Trial/

26 Randomized Controlled Trial/

27 controlled clinical trial/

28 multicenter study/

29 Phase 3 clinical trial/

30 Phase 4 clinical trial/

31 exp RANDOMIZATION/

32 Single Blind Procedure/

33 Double Blind Procedure/

34 Crossover Procedure/

35 PLACEBO/

36 randomi?ed controlled trial$.tw.

37 rct.tw.

38 (random$ adj2 allocat$).tw.

39 single blind$.tw.

40 double blind$.tw.

41 ((treble or triple) adj blind$).tw.

42 placebo$.tw.

43 Prospective Study/

44 or/25-43

45 Case Study/

46 case report.tw.

47 abstract report/ or letter/

48 Conference proceeding.pt.

49 Conference abstract.pt.

50 Editorial.pt.

51 Letter.pt.

52 Note.pt.

53 or/45-52

54 44 not 53

55 24 or 54

56 8 and 11 and 16 and 55

57 exp adult/

58 (adult* or aged or centenarian? or nonagenarian? or octogenarian? or elderly or senior?).mp.

59 57 or 58

60 56 and 59

61 limit 56 to adult

62 60 or 61

63 limit 62 to yr="2016 -Current"

64 limit 63 to (english or french)

**Medline**

1   exp hiv infections/ or acquired immunodeficiency syndrome/ or aids-related opportunistic infections/
2   (advanced HIV disease or hiv infection* or acquired immunodeficiency syndrome or aids-related complex or aids-related opportunistic).mp.
3  (acquired immun* and deficiency syndrome).mp.
4   (Hiv or "hiv‐1" or "hiv‐2*" or "hiv1" or "hiv2" or hiv infect*).mp.
5   ("human immunodeficiency virus" or "human immunedeficiency virus" or "human immuno‐deficiency virus" or "human immune‐deficiency virus").mp.
6   ("acquired immunodeficiency syndrome" or "acquired immunedeficiency syndrome" or "acquired immuno‐deficiency syndrome" or "acquired immune‐deficiency syndrome" or (acquired immun* and deficiency syndrome)).mp.
7   aids.mp.
8   or/1-7

9   mortality/ or "cause of death"/ or fatal outcome/ or hospital mortality/ or mortality, premature/ or survival rate/
10   (mortalit* or death* or dying or survival rate*).mp.
11   9 or 10

12   "sub–Saharan Africa".mp.
13   Subsaharan Africa.mp.
14   exp "Africa South of the Sahara"/
15   (ANGOLA or BENIN or BOTSWANA or BURKINA FASO or CABO VERDE or CAMEROON or CENTRAL AFRICAN REPUBLIC or CHAD or CONGO or COTE D?IVOIRE or DEMOCRATIC REPUBLIC OF THE CONGO or DJIBOUTI or EQUATORIAL GUINEA or ERITREA or ESWATINI or ETHIOPIA or GABON or GAMBIA or GHANA or GUINEA or GUINEA-BISSAU or KENYA or LESOTHO or LIBERIA or MALAWI or MALI or MAURITANIA or MOZAMBIQUE or NAMIBIA or NIGER or NIGERIA or RWANDA or "SAO TOME AND PRINCIPE" or SENEGAL or SIERRA LEONE or SOMALIA or SOUTH AFRICA or SOUTH SUDAN or SUDAN or TANZANIA or TOGO or UGANDA or ZAMBIA or ZIMBABWE).mp. [mp=title, book title, abstract, original title, name of substance word, subject heading word, floating sub-heading word, keyword heading word, organism supplementary concept word, protocol supplementary concept word, rare disease supplementary concept word, unique identifier, synonyms, population supplementary concept word, anatomy supplementary concept word]
16   or/12-15

17   exp cohort studies/
18   cohort$.tw.
19   controlled clinical trial.pt.
20   epidemiologic methods/
21   limit 20 to yr="1971 - 1988"
22   exp case-control studies/
23   (case$ and control$).tw.
24   17 or 18 or 19 or 21 or 22 or 23

25   Randomized Controlled Trials as Topic/
26   randomized controlled trial/
27   Random Allocation/
28   Double Blind Method/
29   Single Blind Method/
30   clinical trial/
31   clinical trial, phase i.pt.
32   clinical trial, phase ii.pt.
33   clinical trial, phase iii.pt.
34   controlled clinical trial.pt.
35   randomized controlled trial.pt.
36   multicenter study.pt.
37   clinical trial.pt.
38   exp Clinical Trials as topic/
39   or/25-38
40   (clinical adj trial$).tw.
41   ((singl$ or doubl$ or treb$ or tripl$) adj (blind$3 or mask$3)).tw.
42   PLACEBOS/
43   placebo$.tw.
44   randomly allocated.tw.
45   (allocated adj2 random$).tw.
46   40 or 41 or 42 or 43 or 44 or 45
47   (random* or rct or rcts).tw.
48   39 or 46 or 47
49   case report.tw.
50   letter/
51   historical article/
52   Comment/
53   49 or 50 or 51 or 52
54   48 not 53
55   24 or 54
56   8 and 11 and 16 and 55

57   adult/ or aged/ or "aged, 80 and over"/ or centenarians/ or nonagenarians/ or octogenarians/ or frail elderly/ or middle aged/ or young adult/
58   (adult* or aged or centenarian? or nonagenarian? or octogenarian? or elderly or senior?).mp.
59   57 or 58
60   56 and 59
61   limit 56 to ("young adult (19 to 24 years)" or "adult (19 to 44 years)" or "young adult and adult (19-24 and 19-44)" or "middle age (45 to 64 years)" or "middle aged (45 plus years)" or "all aged (65 and over)" or "aged (80 and over)")
62   60 or 61

63   limit 62 to yr="2016 -Current"
64   limit 63 to (english or french)

**Supplement List S2: Risk of bias assessment**

**MODIFIED: NEWCASTLE - OTTAWA QUALITY ASSESSMENT SCALE**

**Mortality in adults with advanced HIV in sub-Saharan Africa**

Note: A study can be awarded a maximum of one star for each numbered item, except Comparability (2 stars possible)

**Selection**

1) Representativeness of trial population

a) truly or at least somewhat representative of average adult people living with advanced HIV disease in sub-Saharan Africa ****

b) selected group of users e.g. health care workers only, men/women only, specific age groups CD4 inclusion different to WHO

c) no description of the derivation of the cohort

2) Selection of the cohort

a) all participants derive from the same study/setting ****

b) CD4 strata are drawn from a different source (e.g., <200 outpatients, <100 inpatients)

c) enrolment period >5 years

d) no description of the derivation of the selected cohort

3) Ascertainment of timing/result of CD4 testing

a) CD4 test result available within 30 days of enrolment ****

b) More than 85% of the patient population was ART naïve, or CD4 result was eligibility criteria for the study ****

c) self-reported or test/medical record >30 days prior to inclusion

d) no description

4) CD4 strata

a) All participants are allocated to a CD4 strata <200 cells/mm^3^****

b) No clearly defined CD4 stratum of <200 cells/mm^3^

**Comparability**

1) Comparability of cohorts based on co-intervention

a) >90% of participants started ART within 8 weeks of study start, or ART was part of study intervention, or ART start was an eligibility criterion ** **

b) >90% were on CTX at baseline or within 4 weeks/1 month of study start ****

c) no description

**Outcome**

1) Assessment of outcome

a) death certificates, medical record ****

b) reported by family/community health care workers ****

c) self-report , only phone contact

d) no description

2) Was follow-up long enough for outcomes to occur

a) yes (at least 2 weeks) ****

b) no

3) Adequacy of follow up of cohorts

a) complete follow up of all patients ****

b) subjects lost to follow up unlikely to introduce bias - small number lost - > 98 % follow up, or reasonable description provided of those lost ****

c) follow up rate < 98% and no description of those lost

d) no statement
